# Fluorescent Tracking of Yeast Division Clarifies the Essential Role of Spleen Tyrosine Kinase in the Intracellular Control of *Candida glabrata* in Macrophages

**DOI:** 10.3389/fimmu.2018.01058

**Published:** 2018-05-16

**Authors:** Zeina Dagher, Shuying Xu, Paige E. Negoro, Nida S. Khan, Michael B. Feldman, Jennifer L. Reedy, Jenny M. Tam, David B. Sykes, Michael K. Mansour

**Affiliations:** ^1^Division of Infectious Disease, Massachusetts General Hospital, Harvard Medical School, Boston, MA, United States; ^2^Biomedical Engineering and Biotechnology, University of Massachusetts Medical School, Worcester, MA, United States; ^3^Division of Pulmonary and Critical Care, Massachusetts General Hospital, Boston, MA, United States; ^4^Center for Regenerative Medicine, Massachusetts General Hospital, Boston, MA, United States

**Keywords:** *Candida*, *Candida glabrata*, macrophages, spleen tyrosine kinase, phagosome, carboxyfluorescein succinimidyl ester

## Abstract

Macrophages play a critical role in the elimination of fungal pathogens. They are sensed *via* cell surface pattern-recognition receptors and are phagocytosed into newly formed organelles called phagosomes. Phagosomes mature through the recruitment of proteins and lysosomes, resulting in addition of proteolytic enzymes and acidification of the microenvironment. Our earlier studies demonstrated an essential role of Dectin-1-dependent activation of spleen tyrosine kinase (Syk) in the maturation of fungal containing phagosomes. The absence of Syk activity interrupted phago-lysosomal fusion resulting in arrest at an early phagosome stage. In this study, we sought to define the contribution of Syk to the control of phagocytosed live *Candida glabrata* in primary macrophages. To accurately measure intracellular yeast division, we designed a carboxyfluorescein succinimidyl ester (CFSE) yeast division assay in which bright fluorescent parent cells give rise to dim daughter cells. The CFSE-labeling of *C. glabrata* did not affect the growth rate of the yeast. Following incubation with macrophages, internalized CFSE-labeled *C. glabrata* were retrieved by cellular lysis, tagged using ConA-647, and the amount of residual CFSE fluorescence was assessed by flow cytometry. *C. glabrata* remained undivided (CFSE bright) for up to 18 h in co-culture with primary macrophages. Treatment of macrophages with R406, a specific Syk inhibitor, resulted in loss of intracellular control of *C. glabrata* with initiation of division within 4 h. Delayed Syk inhibition after 8 h was less effective indicating that Syk is critically required at early stages of macrophage–fungal interaction. In conclusion, we demonstrate a new method of tracking division of *C. glabrata* using CFSE labeling. Our results suggest that early Syk activation is essential for macrophage control of phagocytosed *C. glabrata*.

## Introduction

The fungal pathogen *Candida glabrata* is a common cause of invasive bloodstream infections in immunocompromised patients (1). With the rise of organ transplantation, immunosuppressive chemotherapy, implanted medical devices, and antibacterial use, *C. glabrata* has emerged as a frequently isolated species, accounting for 20% of systemic bloodstream *Candida*-related infections in North America (2, 3).

*In vitro*, following interaction with human or murine macrophages, *C. glabrata* undergoes phagocytosis. Despite being internalized, *C. glabrata* survives and replicates inside host immune cells (4, 5). Intracellular killing of *C. glabrata* by macrophages is only about 10% effective (6). The capacity of this *Candida* species to evade the innate immune system complicates evaluation of intracellular pathogen control by simple phagocytosis assays. A more sensitive and accurate approach to quantifying *C. glabrata* intracellular proliferation is required to understand and identify the relevant pathogen–host interactions.

The carboxyfluorescein diacetate succinimidyl ester (CFSE) assay is a well-established method to fluorescently track lymphocyte cellular division (7, 8). The succinimidyl moiety of the dye covalently attaches to cellular aliphatic amine groups, forming stable amide bonds (9). This covalent-coupling dye allows for tracking of lymphocyte proliferation; each progressive cell division results in a twofold dilution of the fluorescent signal between daughter cells (10, 11). Unlike labeling techniques that rely on microscopic evaluation, CFSE can be visualized by both fluorescence microscopy and can be formally quantitated by flow cytometry (12). CFSE is highly fluorescent, has an excitation/emission profile at 491/518 nm, is stable in long-term tracking, and can be used *in vitro* and *in vivo* (10).

Although most published reports have used CFSE to monitor lymphocyte proliferation, recent studies have also used the dye to follow proliferation of other mammalian and non-mammalian cell types, such as bacterial cells and parasites (13, 14). CFSE also successfully labeled *Paracoccidioides* yeast in a flow cytometry based phagocytosis study, although the tracking of proliferation was not evaluated (15).

Here, we have applied the CFSE proliferation-tracking properties to *C. glabrata*, though several factors require special consideration. Unlike animal cells, fungi possess a cell wall composed of extracellular carbohydrate-rich polymers. CFSE brightly labels the cell wall, though does not permeate the yeast to label intracellular structures. Additionally, unlike mammalian cells where the cell membrane is shared symmetrically (in equal amounts) between daughter cells (16), the yeast cell wall is shared asymmetrically, with the budding yeast generating an almost-entirely new cell wall (17). This new (and CFSE-unlabeled) cell wall is synthesized at growing cell tips through the localization and activation of cell wall carbohydrate synthases (18, 19). Thus, unlike CFSE assays in lymphocytes, the CFSE labeling of yeast such as *C. glabrata* does not allow one to track beyond a single division.

Following infection, tissue macrophages sense fungi through the expression of pattern-recognition receptors (PRRs), such as Dectin-1 and Dectin-2, which recognize fungal cell wall carbohydrates (5, 20). Recognition through PRRs triggers phagocytosis, engulfing microorganisms within phagosomes, eventually fusing with additional subcellular compartments including lysosomes for acidification and killing (21). The process of acidification is termed phagosomal maturation, which in response to fungal cell wall ligands, is spleen tyrosine kinase (Syk)-dependent (22). Syk activation and signal transduction is central for immune activation in several immune cells (23). PRRs share the ability to associate with immunoreceptor tyrosine-based activation motifs (ITAM) in their cytoplasmic domains, leading to Syk recruitment and phosphorylation, resulting in downstream events including phagocytosis, and the production of reactive oxygen species (ROS) and proinflammatory cytokines (24–26). The central role of Syk in activation of several innate and adaptive immune cells has made this kinase a promising target for the development of anti-inflammatory therapeutics (27, 28).

Although the role of Syk in intracellular signaling has been studied extensively, its involvement in the response to intracellular pathogens in innate immune cells, such as macrophages, is not delineated (23, 26). In this study, we introduce a CFSE-labeling assay that allows for intracellular tracking of yeast proliferation in macrophage phagosomes. We demonstrate that CFSE is a reliable method of evaluating *C. glabrata* division by fluorescence microscopy and quantitatively by flow cytometry. Employing the CFSE assay, we show that Syk inhibition in macrophages results in the loss of the ability to control *C. glabrata* proliferation. This loss of *C. glabrata* division control is independent of phagocytosis and is not a result of failure to activate the Dectin-1 and Dectin-2 pathways. These observations highlight CFSE live labeling as a useful and convenient tool for measuring yeast proliferation accurately and support further investigation into the role of Syk in antifungal immunity.

## Materials and Methods

### Reagents

R406 was purchased from Santa Cruz Biotechnology (Dallas, TX, USA), Nonidet P40 (NP40) and peptone from American Bioanalytical (Natick, MA, USA), CFSE dye, and all other chemicals from Sigma (St. Louis, MO, USA) unless otherwise stated. Wild type *C. glabrata* (ATCC2001) was purchased from the American Type Culture Collection (ATCC, Manassas, VA, USA). Cell lysis buffer consisted of 4× NP40 containing 40 mM Tris–hydrochloric acid, 600 mM sodium chloride, 20 mM magnesium chloride, and 4% NP40 titrated to pH 7.5. Yeast culture media liquid YPD contained 1% yeast extract (BD Biosciences, San Jose, CA, USA), 2% peptone, and 2% dextrose. Cell culture media (RPMI-complete) was composed of RPMI 1640 (Corning, Tewksbury, MA, USA) with 2 mM l-glutamine, 10% heat-inactivated fetal bovine serum, and 1% penicillin–streptomycin (ThermoFisher Scientific, Waltham MA, USA).

### Yeast Culture and CFSE Staining

*Candida glabrata* were grown overnight shaking in liquid YPD at 30°C, washed three times in phosphate buffered saline (PBS), counted using a Luna automated cell counter (Logos Biosystems, Annandale, VA, USA), and resuspended in PBS at the desired inoculum.

To stain yeast with CFSE, wild type *C. glabrata* were grown to log phase in liquid YPD, washed twice in 1 mL PBS, and 160 million yeast were stained with 25 µg/mL CFSE for 30 min in 4 mL PBS at 30°C on a vertical rotator. Yeasts were washed twice in 10 mL PBS containing 2% bovine serum albumin (BSA) to remove excess CFSE. Stained yeast cells were resuspended in 1 mL PBS and passed through a 25G 7/8-inch needle 10 times to dissociate clumps into single cell suspension.

### Calcofluor White Staining

To stain all intracellular yeast cells, macrophages were fixed and permeabilized with 4% paraformaldehyde (Electron Microscopy Sciences, Hatfield, PA, USA) for 20 min at room temperature in the dark. Cells were washed three times with PBS and incubated in 10% calcofluor white solution in PBS for 15 min and imaged using confocal microscopy with 405 nm laser excitation.

### Collection of Murine Peritoneal Macrophages

Eight-week-old inbred C57BL/6 mice (Jackson Laboratory, ME, USA), Dectin-1^−/−^ (B6 background, a gift from Dr. Gordon Brown, University of Aberdeen, UK) (29), or Dectin-2^−/−^ (B6 background, a gift from Dr. Marcel Wuethrich, University of Wisconsin) (30) were housed in a specific pathogen-free facility at the Massachusetts General Hospital (MGH, Boston, MA, USA). All animal experiments were approved by the MGH Institutional Animal Care and Use Committee. Peritoneal macrophages were isolated from mice following intraperitoneally instillation of 2 mL thioglycollate (Northeast Laboratory, Waterville, ME, USA). After 3 days, the mice were euthanized and the peritoneum lavaged with 10 mL RPMI-complete to retrieve cells. Intraperitoneal cells were washed and plated at a density of 300,000 cells per well in 500 µL RPMI-complete in 24-well dishes and allowed to adhere overnight. Non-adherent contaminating cells were washed away after 24 h resulting in a >95% pure, viable macrophage population as determine by anti-Mac 1 staining using flow cytometry (data not shown).

### Co-Culture of Peritoneal Macrophages and CFSE-*C. glabrata*

The media in each 24-well was reduced to 250 µL and wells were treated with R406 dissolved in DMSO or DMSO only either 10 min prior to *C. glabrata* inoculation or 2, 4, or 8 h after *C. glabrata* inoculation. A total of 200,000 CFSE-labeled *C. glabrata* were added for a final MOI of 0.6. Plates were centrifuged for 1 min at 700 × *g* to facilitate cell-ligand contacts and incubated at 37°C with 5% CO_2_. After the time indicated, the macrophages were lysed with 100 µL of 4× NP40 on ice for 5 min. Well contents were centrifuged for 7.5 min at 10,000 rpm. The pellets were then fixed with 4% formaldehyde for 10 min in the dark and washed with PBS/2% BSA. Concanavalin A conjugated to Alexa Fluor 647 (ThermoFisher, Waltham, MA, USA) at a final concentration 3 µg/mL was added for 20 min in the dark to stain *C. glabrata*. Yeast were washed and resuspended in PBS/2% BSA for flow cytometry.

### Macrophage Phagocytosis of *C. glabrata*

To assess macrophage phagocytosis, CFSE-*C. glabrata* were co-incubated with macrophages in the presence or absence of inhibitor for the time indicated. Plates were transferred onto ice, wells washed with PBS, then macrophages mechanically lifted, and strained through a 40-μm filter and immediately subjected to flow cytometry gating on the macrophage population. The phagocytosis percent was measured as the number of CFSE positive macrophages over the total macrophage cell number.

### Reactive Oxygen Species

Reactive oxygen species was measured as described (31), briefly macrophages were plated at 5 × 10^4^ cells/well in white wall 96-well plate (Costar, Cambridge, MA, USA). Cells were placed on ice and washed three times with PBS. Lucigenin solution (0.9 mM CaCl_2_, 0.5 mM MgCl_2_, 20 mM dextrose, and 20 µM lucigenin) was added, and cells were incubated on ice for 10 min. Heat-killed *C. glabrata* for 10 min were added at an effector to target ratio of 25, and then the plate was centrifuged at 750 × *g* for 1 min to facilitate cell-ligand contacts. An initial reading was then taken immediately after centrifugation for baseline. The plate was then incubated at 37°C and read with SpectraMax i3x reader (Molecular Devices, Sunnyvale, CA, USA) for total luminescence every 10 min for 2 h.

### Confocal Microscopy

2 × 10^4^ peritoneal macrophages were plated onto eight-chambered coverslip slides (LabTek, Thermo Scientific, Rochester, NY, USA) in RPMI-complete. CFSE-labeled *C. glabrata* were added at MOI 1:1, spun at 700 *g* × 1 min for immediate co-culture and slides mounted on a Nikon Ti-E inverted microscope equipped with an EM-CCD camera (Hamamatsu Photonics K.K., Hamamatsu, Japan). The excitation source was an 89 North MultiLine LaserBank (89 North, Burlington, VT, USA) A piezo stage (Prior Instruments, Rockland, MA, USA) capable of *X, Y*, and *Z* movement was used for acquisition. A polarizer (Nikon, MEN 51941) and Wollaston prisms (Nikon, MBH76190) were used to acquire differential interference contrast (DIC) images. Emission light from the samples was collected after passage through the appropriate emission filters (Semrock, Rochester, NY, USA) (32). Images were acquired using MetaMorph software (Molecular Devices, Downingtown, PA, USA), and processed using Adobe Photoshop CS5 and assembled in Adobe Illustrator, version CS5 (Adobe Systems, San Jose, CA, USA).

### Flow Cytometric Analysis of CFSE

Carboxyfluorescein succinimidyl ester-labeled *C. glabrata* that were fixed and stained with concanavalin A-Alexa Fluor 647 were flowed using FACS Calibur flow cytometer (Becton-Dickinson, San Jose, CA, USA) and CellQuest software (Becton-Dickinson). ConA-647 (FL4) was plotted on the *y*-axis and CFSE (FL1) on the *x*-axis and the percentage of CFSE bright undivided population was determined with FlowJo 10 (FlowJo, Ashland, OR, USA).

### Statistics

Statistical calculations were performed using GraphPad Prism 7 software. Data were analyzed by two-tailed, unpaired *t* test and were considered significantly different when *p* ≤ 0.05.

## Results

### CFSE Labeling Tracks Division of *C. glabrata*

**Figure 1:**
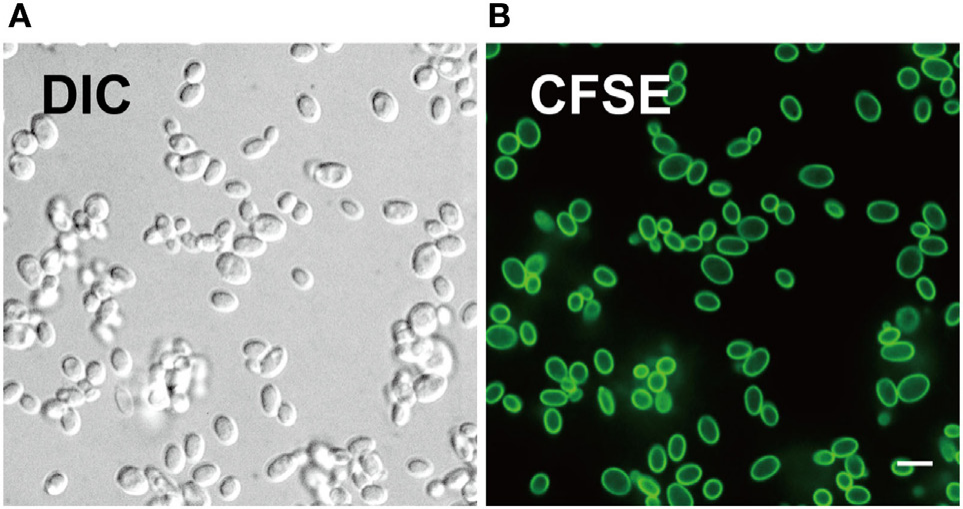
Carboxyfluorescein succinimidyl ester (CFSE) labels cell wall of *Candida glabrata*. **(A)** Differential interference contrast and **(B)** fluorescence confocal microscopy image of live *C. glabrata* stained with CFSE showing labeling of all yeast cells prior to cell division. Scale bar represents 5 µm.

**Figure 2:**
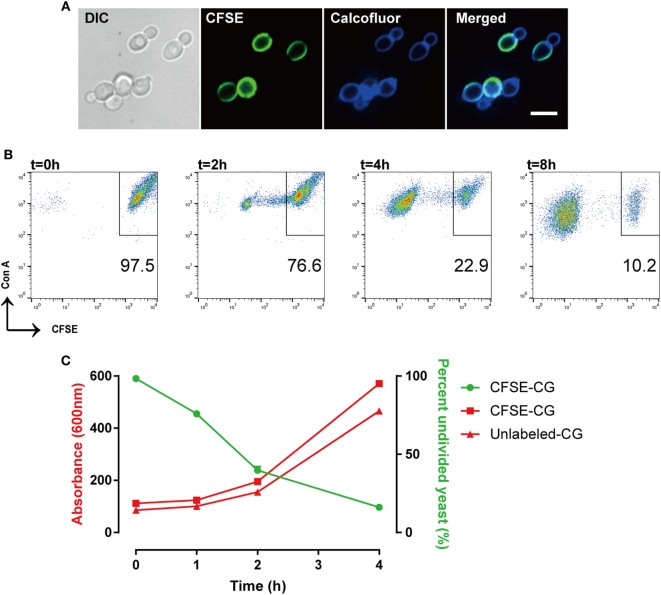
Carboxyfluorescein succinimidyl ester (CFSE)-labeling marks *Candida glabrata* yeast division. **(A)** Differential interference contrast and fluorescence microscopy image of CFSE-labeled *C. glabrata* that have undergone division and stained with calcofluor white. Parental cells exhibit full CFSE labeling, while daughter cells have diluted CFSE labeling leading to dimmer fluorescence signal. Scale bar represents 5 µm. **(B)** Scatter plot of Con A-AF647 and CFSE fluorescence. CFSE-bright parental yeast population in upper right quadrant gate. Percent (scatter plot inset) of undivided CFSE-bright population was determined over time indicated. **(C)** Correlation of percent CFSE-bright population to OD_600_ nm of proliferating yeast. Data represent a minimum of three independent triplicate experiments.

We evaluated CFSE staining of *C. glabrata* using fluorescence microscopy. *C. glabrata* were homogenously fluorescent throughout the cell wall, likely due to the high content of fungal cell wall esterases that permit CFSE activation and labeling. The CFSE-labeled *C. glabrata* retained normal cell shape and morphology (Figure 1). Following 6 h in culture, CFSE-labeled *C. glabrata* were counter-stained with calcofluor white for chitin to delineate all yeast cells and visualized using confocal microscopy. All yeast cells were evenly stained with calcofluor white. Parent yeast cells were brightly CFSE-fluorescent, while daughter cells exhibited diluted CFSE leading to reduced fluorescence intensity as identified by flow cytometry (Figure 2A).

The consistent and predictable decrease in fluorescence permits quantitation of *C. glabrata* intracellular division as a marker of control by macrophages. While calcofluor allows visualization of all yeast cells using 405 nm laser excitation under confocal microscopy, our FACSCalibur is fitted with a blue and red laser incapable of exciting calcofluor. For these reasons, the total number of yeast were identified by labeling with concanavalin A conjugated to AF647 for flow cytometry. Therefore, a *C. glabrata* parent cell population is double-positive for CFSE and ConA-647, while divided daughter cells are CFSE-dim, but remain ConA-647 (Figure 2B).

With each cell division, the percent of the parent cells decrease, as daughter cell numbers rise and are easily distinguished *via* CFSE intensity. In fact, the diminished CFSE-bright parent cells was inversely proportional to the increase in the optical density of the growing yeast, confirming that the percentage of CFSE-bright cells accurately reflects yeast proliferation (Figure 2C). Moreover, CFSE-labeled and unlabeled *C. glabrata* proliferated at the same rate (as measured by OD_600_) indicating that the CFSE-labeling process does not affect yeast proliferation.

### Macrophages Control Intracellular *C. glabrata* Proliferation Using Syk

**Figure 3:**
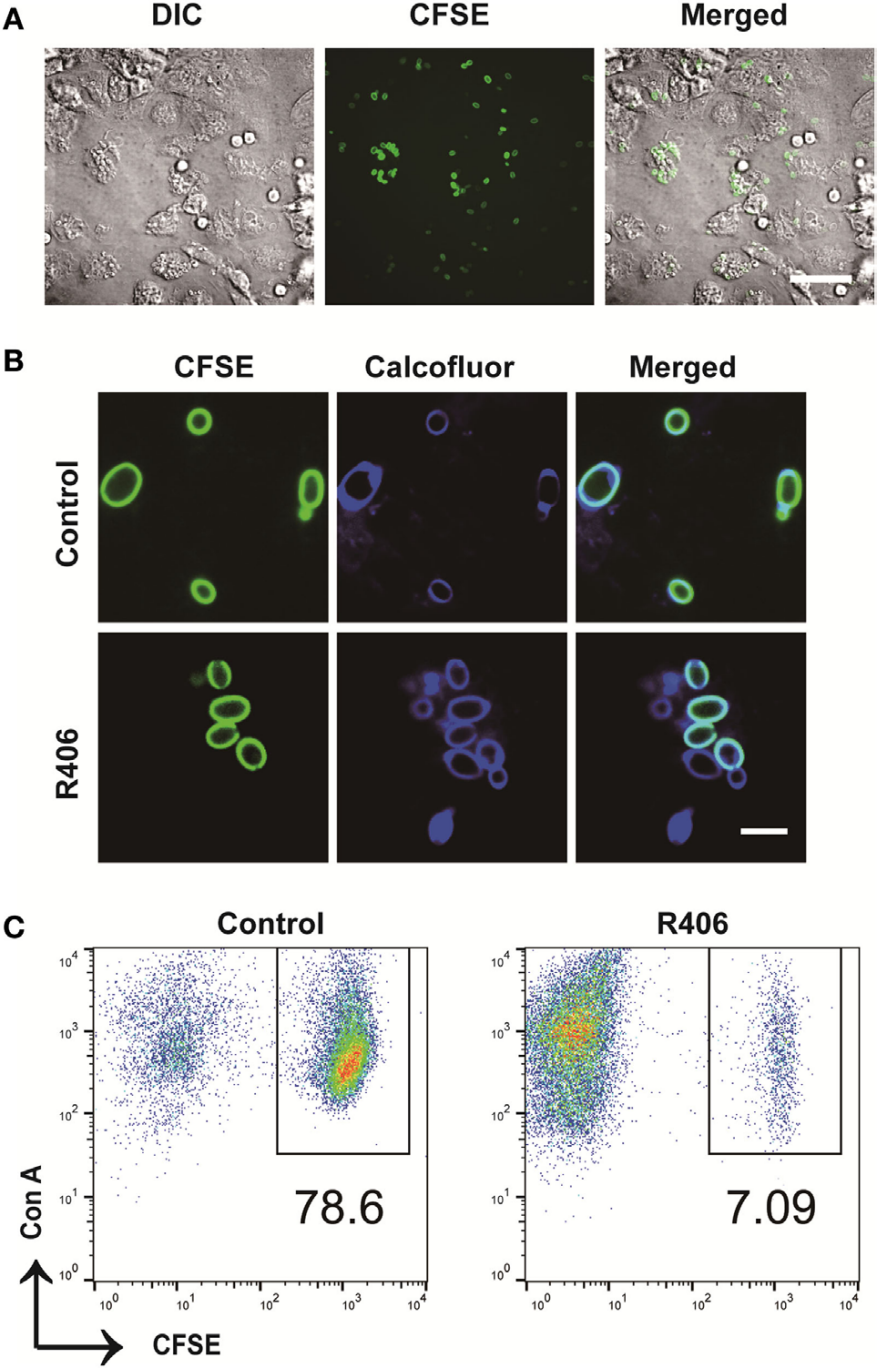
Spleen tyrosine kinase is required for peritoneal macrophage control of *Candida glabrata*. **(A)** Differential interference contrast (DIC) and fluorescence microscopy image of carboxyfluorescein succinimidyl ester (CFSE)-*C. glabrata* and macrophage after 1 h of coculture indicating complete uptake of the yeast by the macrophages. Scale bar represents 50 μm. **(B)** DIC and fluorescence microscopy image of CFSE *C. glabrata* infected macrophages 16 h post inoculation. Macrophages control *C. glabrata* division in CFSE-bright undivided state, while treatment with R406 impaired the macrophages’ ability in controlling *C. glabrata*. Scale bar represents 5 µm. **(C)** Flow cytometry scatter plots of Con A and CFSE showing macrophage controlling *C. glabrata* division and loss of control in R406-treated cells. Data represent three independent experiments.

To evaluate the role of Syk in macrophages following exposure to *C. glabrata*, we used an *in vitro* co-culture system. Peritoneal macrophages were co-cultured with CFSE-labeled *C. glabrata* parent cells and monitored for evidence of intracellular proliferation. In wild type murine macrophages, yeast cells were phagocytosed by macrophages with high efficiency. There were rare numbers of extracellular yeast remaining as measured by microscopy (Figure 3A) and flow cytometry analysis of co-culture supernatant (data not shown). Following co-culture incubation, analysis of CFSE intensity of *C. glabrata* from lysed macrophages revealed that yeast did not divide for up to 16 h as measured by retained parent CFSE-bright (Figure 3B, top panel). However, in the presence of the reversible Syk inhibitor, R406, macrophages were unable to control *C. glabrata* division (Figure 3B bottom panel). Compared to untreated controls, R406-treated macrophages resulted in a diminished number of parent yeast and in a growing numbers of divided daughter population. While these yeast remained intracellular, they had escaped control and undergone division as noted by loss of CFSE (Figure 3C). To ensure that R406 does not alter growth kinetics of *C. glabrata*, division rates of *C. glabrata* in the presence of R406 and was not different from yeast control conditions (data not shown). These data suggest that loss of Syk activity significantly impaired the ability of macrophages to control *C. glabrata* intracellular division.

### Early Syk Activity Is Required for Intracellular *C. glabrata* Control

**Figure 4:**
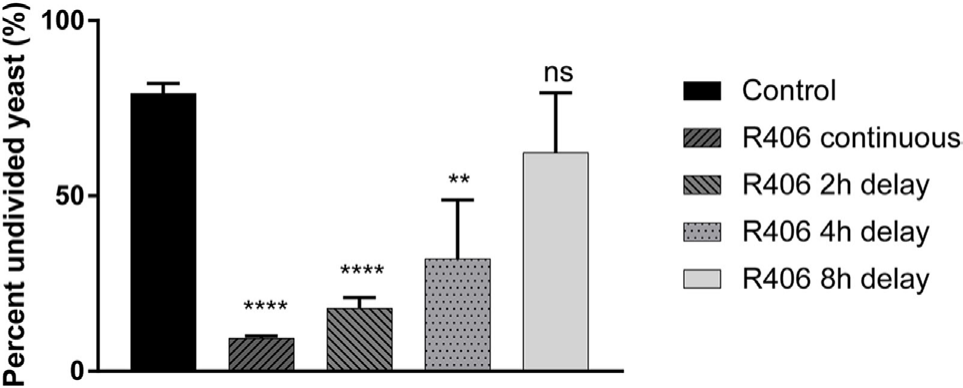
Spleen tyrosine kinase (Syk) is required at an early stage for macrophage control of *Candida glabrata*. Macrophages were treated with chemical Syk inhibitor, R406, prior to addition of *C. glabrata* for 2, 4, and 8 h after infection. All wells were lysed after 16 h of co-culture and the yeast retrieved and stained with ConA-AF647. Percent of parental CFSE-bright population was assessed *via* flow cytometry, with R406 treatments initiated at indicated time. **p ≤ 0.01, ***p ≤ 0.001, ****p ≤ 0.0001. Data represent a minimum of three independent experiments.

Given that Syk is essential for control of *C. glabrata* division, we next sought to define the temporal relationship between Syk activity and yeast control. Macrophages that had engulfed *C. glabrata* were exposed to R406 continuously or following a 2, 4, or 8-h delay from time of coincubation. Syk inhibition at the start, or 2- or 4-h resulted in a loss of macrophage control as evidenced by the significant decrease in the percent of undivided yeast (Figure 4). The delayed addition of R406 to 8 h, and beyond, did not have an influence *C. glabrata* division. These data suggest that it is the early Syk activity following macrophage phagocytosis of yeast that is required for intracellular *C. glabrata* control.

### Syk Inhibition Does Not Affect Macrophage Phagocytosis

In certain cell types, Syk has been shown to play a role in cytoskeletal rearrangement and endocytosis (33). If the process of macrophage phagocytosis was impaired by Syk inhibition (R406), this might leave extracellular *C. glabrata* in an actively dividing state. To determine if Syk inhibition impacts the process of phagocytosis of *C. glabrata* by peritoneal macrophages, we measured phagocytosis by flow cytometry in the presence and absence of R406. Cytochalasin D, an actin polymerization inhibitor, was used as a positive control inhibitor of phagocytosis.

**Figure 5:**
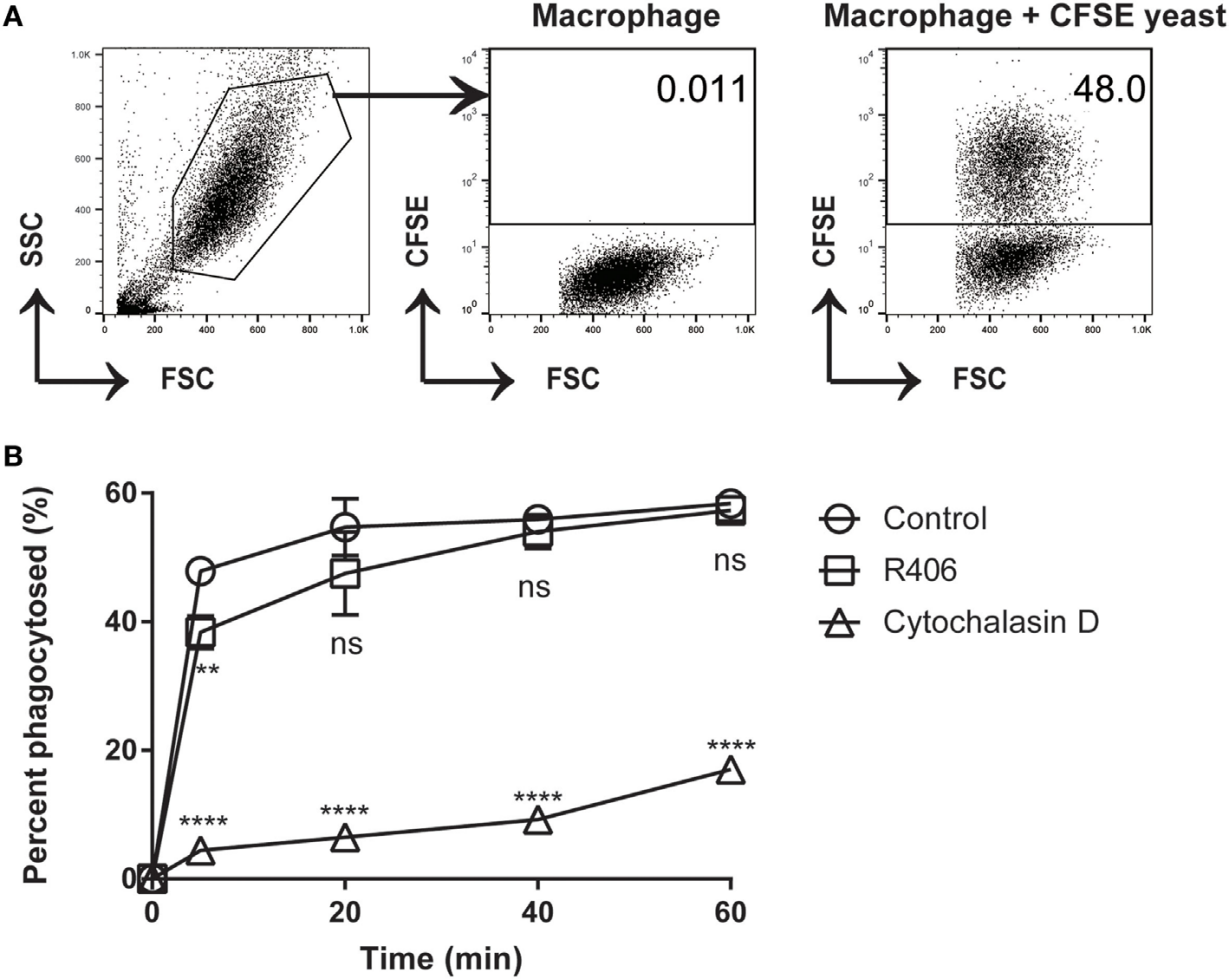
R406-induced spleen tyrosine kinase inhibition does not affect macrophage phagocytosis of *C. glabrata*. **(A)** Gating scheme for calculated percentage of macrophages that have phagocytosed CFSE-labeled *C. glabrata* by flow cytometry. **(B)** Percent phagocytosis of *C. glabrata* by macrophages over time incubated with control, R406, or cytochalasin D by flow cytometry. **p ≤ 0.01, ****p < 0.0001. Data represent three independent experiments.

We first determined the percent of macrophages that were associated with CFSE-bright *C. glabrata*. There was no difference in the ability of macrophages to phagocytose *C. glabrata* in the presence of R406 after 5 min (Figure 5). Macrophages that were treated with cytochalasin D showed dramatically reduced phagocytosis. The small percentage of yeast associated with cytochalasin D-treated macrophages as detected by flow possibly represents CFSE-bright *C. glabrata* attached extracellularly to the macrophage membrane or incomplete inhibition of phagocytosis by cytochalasin D. These findings indicate that Syk plays a minor role in the process of macrophage phagocytosis of *C. glabrata*.

### R406 Inhibition of Syk Does Not Result in Permanent Macrophage Impairment

Given that Syk-mediated pathways are involved in multiple macrophage functions, we sought to determine if chemical Syk inhibition might result in a persistent hyporesponsive state. Given that ROS is a key mechanism in the response of macrophage to fungi, we evaluated ROS production in primary macrophages treated with R406 as compared to macrophages pulsed with R406 and then washed free of the inhibitor.

**Figure 6:**
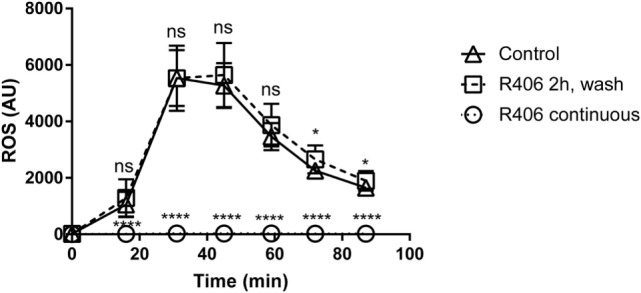
R406 does not result in permanent macrophage response defects to fungal stimuli as measured by reactive oxygen species (ROS) production. Plated macrophages were treated with R406 for 2 h, and then washed to remove the inhibitor. ROS production measured by lucigenin after addition of heat-killed *Candida*. Arbitrary light unites (AU) represents total luminescence corresponding with ROS production. *p ≤ 0.05, ****p ≤ 0.0001. Data represent a minimum of three independent experiments.

With constant R406, ROS production was completely suppressed (Figure 6). However, removal of R406 (following 2 h of exposure) restored ROS production to baseline levels. The observation that R406-pulsed macrophages were capable of generating robust ROS mirroring those of untreated control cells suggests that inhibition of Syk does not permanently alter the viability or biological activity of macrophages.

### Dectin-1 and Dectin-2-Dependent Syk Activity Are Dispensable for Control of Intracellular *C. glabrata* Growth

**Figure 7:**
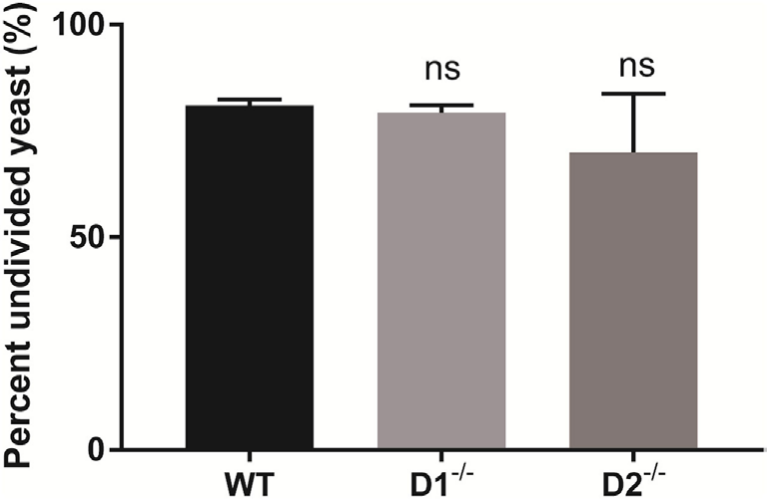
Spleen tyrosine kinase-mediated control of *Candida glabrata* is independent of Dectin-1 and Dectin-2. Percent undivided carboxyfluorescein succinimidyl ester-bright *C. glabrata* population from peritoneal macrophages-yeast co-cultures. Macrophages were obtained from wild type, Dectin-1^−/−^, and Dectin-2^−/−^ mice. Comparisons between all groups are non-significant. Data represent a minimum of three independent experiments.

Dectin-1 and Dectin-2 are upstream carbohydrate lectin PRRs that rely on Syk to regulate intracellular macrophage responses. To determine if the Syk activity required for intracellular *C. glabrata* control was derived through activation of Dectin-1 or Dectin-2, we compared peritoneal macrophages derived from wild type, to those derived from Dectin-1-deficient and Dectin-2-deficient mice. Following macrophage co-culture with CFSE-labeled *C. glabrata*, there was no difference in the control of intracellular *C. glabrata* growth between the macrophage genotypes signifying that the critical Syk activity required for yeast control is not related to individual Dectin-1 or Dectin-2 function (Figure 7).

## Discussion

Though the importance of the innate immune system in protecting against fungal pathogens has been articulated, defining the mechanisms responsible for the intracellular control of *C. glabrata* have yet to be defined. In this study, we define the role of Syk in the intracellular control of *C. glabrata* division in macrophages. Our approach outlines the CFSE-labeling technique as a new tool to track yeast division. We demonstrate that in macrophages, early Syk activity is essential for controlling *C. glabrata* division, and that this role is not solely dependent on the contribution of Dectin-1 or Dectin-2, both of which rely principally on Syk for intracellular signaling following ligand engagement.

We delineated a method of CFSE labeling that allows tracking of yeast multiplication. This process involved conjugating the cell wall of *C. glabrata* cells with CFSE, a fluorescent dye that links to amine groups through covalent bonds, and that can be visualized using confocal microscopy and flow cytometry. Microscopy demonstrated that CFSE does not change the morphology of *C. glabrata* cells. CFSE stained only the cell walls of parent yeasts, which permitted us to distinguish CFSE-bright parent populations and CFSE-dim daughter populations. The increase of CFSE-dim yeast cells, indicative of cell division and a growing daughter population, could be accurately measured by flow cytometry.

While CFSE assays have historically been used to follow the division of lymphocytes, our adaptation to track yeast division does face limitations. CFSE stains only the fungal cell wall rather than the contents of the cytoplasm, which allows us to track only a single generation of daughter cells. This observation is likely due to the presence of the esterase-rich fungal cell wall. Esterases are necessary for the activation of CFSE for covalent conjugation to amino groups on proteins. In mammalian cells, esterases are abundant within the cytoplasm, whereas in yeast, high levels of esterases are found within the fungal cell wall (34). This esterase distribution clarifies the reason for concentrated CFSE conjugation to the cell wall ultrastructure as opposed to homogenous cytoplasmic staining as seen in lymphocytes. Given the high concentration of CFSE in the cell wall, we confirmed that labeling did not alter the fungal cell wall or yeast division. By monitoring growth *via* optical density at 600 nm, we show that CFSE-labeled *C. glabrata* are capable of proliferating with normal kinetics as compared to unlabeled controls. While this method allows tracking of intracellular yeast division, it does not confirm killing of the yeast by macrophages.

Using CFSE-labeled yeast, we defined the role of Syk in the intracellular control of *C. glabrata* by macrophages. We hypothesized that Syk is indeed necessary for macrophages to maintain growth control of *C. glabrata* given its described contribution to phagolysosomal maturation (22) and autophagy (31). The downstream Syk-dependent molecular mechanisms critical for control of *C. glabrata* will require additional investigation and may involve sequestration of essential nutrients or disrupted fusion of lysosomal content. The C-type lectin receptors, Dectin-1 and Dectin-2, recognize fungi and are linked through Syk for intracellular signaling (35). We determined the contribution of each receptor to Syk activity in macrophages following *Candida* exposure. Data reveal that loss of either receptor does not impact the control of intracellular *C. glabrata* suggesting that Syk activity is stimulated through redundant pathways.

In addition to Syk being important for *C. glabrata* control, we also demonstrates the importance of timing of Syk activity. Early Syk activation, within the first 4 h after macrophage phagocytosis *C. glabrata*, is the essential window for effective control of *C. glabrata* division. Macrophages in which Syk was inhibited 2 or 4 h after addition of *C. glabrata* enabled the yeast to divide unchecked. Syk may have less relevance when inhibited 8 h after co-infection because the macrophages retain their ability to control yeast division in similar fashion to untreated macrophages. These observations may be due to time-dependent phagosomal mechanisms, which have no further consequence on *C. glabrata* division at later stages following phagocytosis. In addition, *C. glabrata*-specific immune evasion pathways may be upregulated at later time points, and these may bypass Syk-related control mechanisms. In this study, we have only evaluated chemical Syk inhibition, and have yet to test Syk knockout models.

Spleen tyrosine kinase has an established role in cytoskeletal changes, which could potentially impact phagocytosis. To control for this possibility, we measured uptake of *C. glabrata* by macrophages and found no difference in phagocytosis between control and R406-treated macrophages. This observation is in line with our previous observation using tyrosine mutations in the hemi-ITAM of Dectin-1, which result in complete loss of Syk phosphorylation, yet maintains near normal rates of phagocytosis (22). In addition, to address the likelihood of a lasting effect triggered through Syk inhibition, we utilized ROS activity as a surrogate marker of macrophage activity. After washout of R406, ROS activity is fully restored, indicating that transient Syk inhibition does not result in a long-lasting inhibition of macrophages, and does not account for loss of intracellular *C. glabrata* activity.

In conclusion, our results demonstrate that macrophages control the division of *C. glabrata* in a Syk-dependent manner. A limitation of this observation is the use of a single ATCC strain and further investigation is required to determine if Syk-dependent control is generalizable to other *Candida* species. These observations were verified by a CFSE-labeling technique that accurately distinguished CFSE-bright parent and CFSE-dim daughter cell populations through flow cytometric analysis or microscopy. We show that Syk in macrophages is essential in the early stages of *C. glabrata* infection and that Dectin-1 or Dectin-2 are not individual contributors to Syk activity. Further investigations are required to define the precise downstream mechanisms of Syk activity in the intracellular control of fungal pathogens.

## Ethics Statement

This study was carried out in accordance with the recommendations of Massachusetts General Hospital Institutional Animal Care and Use Committee. The protocol was approved by the Massachusetts General Hospital Institutional Animal Care and Use Committee.

## Author Contributions

ZD, SX, PN, MM, and DS contributed to experimental design. ZD, SX, PN, JT, and MM executed the experiments. ZD, SX, PN, NK, MF, JR, JT, DS, and MM performed analysis and interpretation of experiments. ZD, SX, PN, and MM contributed in writing the manuscript.

## Conflict of Interest Statement

The authors declare that the research was conducted in the absence of any commercial or financial relationships that could be construed as a potential conflict of interest.
